# Left bundle branch area pacing in children: a systematic review and meta-analysis of feasibility, safety, and outcomes

**DOI:** 10.3389/fcvm.2026.1923714

**Published:** 2026-09-16

**Authors:** Hei-To Leung, Sabrina Tsao, Wilfred Hing-Sang Wong, Yiu-Fai Cheung, Sit-Yee Kwok

**Affiliations:** 1 Cardiology Centre, Department of Paediatrics and Adolescent Medicine, Hong Kong Children’s Hospital, Kowloon Bay, Hong Kong, China; 2 Department of Paediatrics and Adolescent Medicine, School of Clinical Medicine, LKS Faculty of Medicine, The University of Hong Kong, Hong Kong, China

**Keywords:** conduction system pacing, congenital heart disease, left bundle branch area pacing, pediatric, pediatric pacing

## Abstract

**Background:**

Chronic right ventricular pacing in children has been associated with pacing-induced cardiomyopathy. While left bundle branch area pacing (LBBAP) offers a physiological alternative to preserve synchronous activation, its application in the paediatric population involves distinct technical and anatomical challenges

**Objectives:**

To systematically evaluate the procedural feasibility, safety, and electromechanical outcomes of LBBAP in paediatric patients.

**Methods:**

PubMed/MEDLINE, Embase, Scopus, and Cochrane databases were searched using medical subject heading terms “left bundle branch area pacing,” “LBBAP,” “conduction system pacing,” “paediatric,” “children,” “congenital heart disease,” and similar keywords. Random-effects meta-analyses of proportions and means, subgroup analyses, and meta-regressions were conducted.

**Results:**

A total of 127 paediatric patients (≤ 18 years) in 8 cohorts were included. The pooled procedural success rate was 80.0%, with a low aggregate complication rate of 9.7% and satisfactory early-to-intermediate term capture thresholds. Statistical analyses revealed no significant clinical predictors of procedural feasibility. While current data did not identify patient size as a significant limitation, a numerically lower success rate was observed in complex congenital heart disease (CHD) compared to simple CHD (64.0% vs. 83.6%). The mean postprocedural QRS duration and left ventricular ejection fraction showed no significant changes. Notably, among the 50 patients with available echocardiographic data, LBBAP yielded a statistically significant reduction in left ventricular end-diastolic dimension Z-scores (pooled mean difference −1.12; *p* = 0.026).

**Conclusions:**

Preliminary evidence suggests that LBBAP is a feasible physiological pacing strategy in paediatric patients that demonstrates an acceptable safety profile and is associated with reduced left ventricular dimensions. Although the impacts of patient size and complex congenital heart disease on procedural success did not reach statistical significance in this cohort, both factors likely remain notable technical challenges.

**Systematic Review Registration:**

(https://www.crd.york.ac.uk/PROSPERO/view/CRD420261287485) PROSPERO CRD420261287485.

## Graphical Abstract

**Figure:**
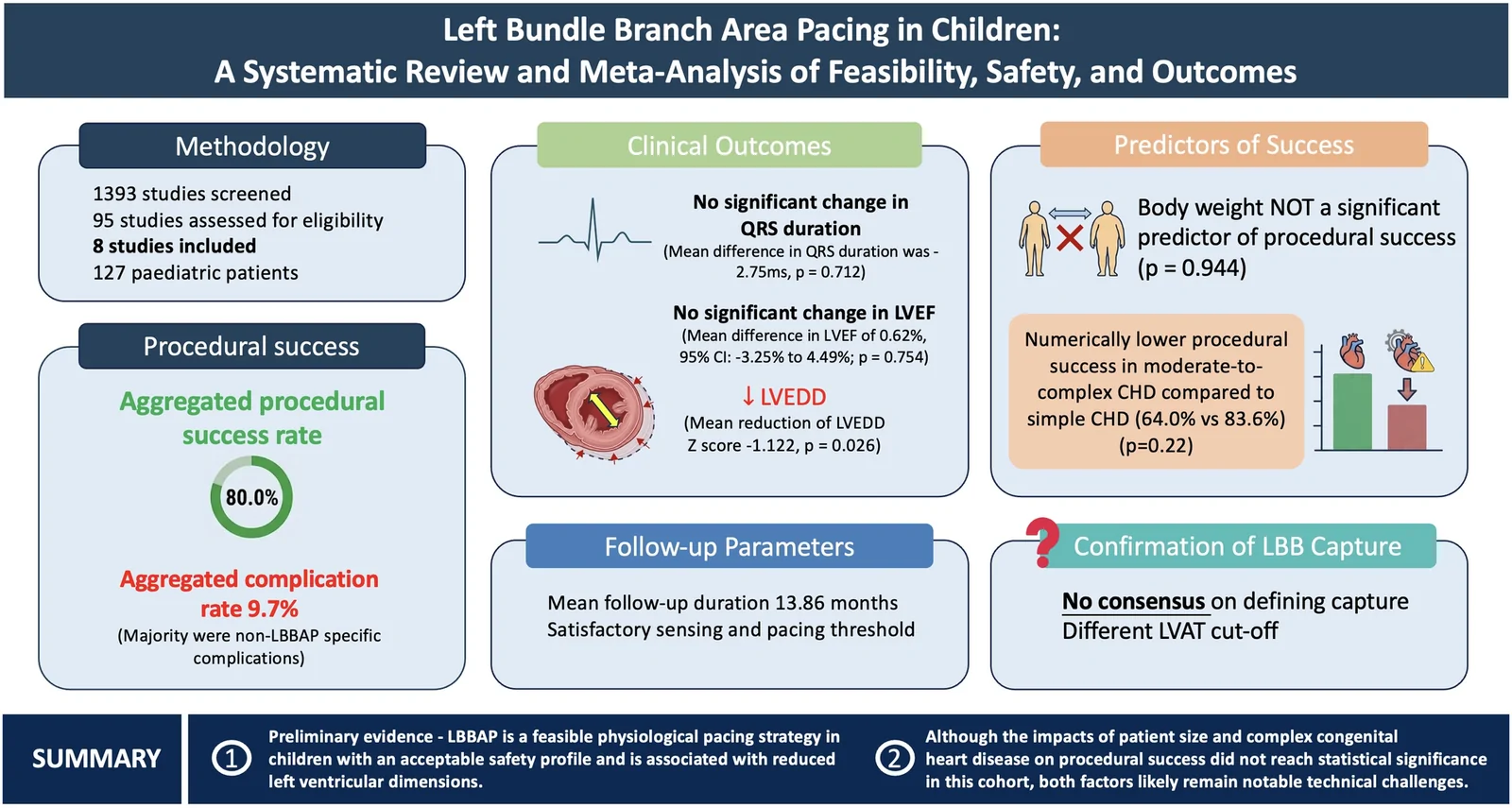

## Introduction

Chronic right ventricular (RV) pacing is historically the standard treatment for paediatric bradycardia and complete atrioventricular block. However, long term RV pacing is associated with significant mechanical and electrical dyssynchrony, potentially contributing to pacing-induced cardiomyopathy (1). Paediatric patients are believed to be more vulnerable to these adverse effects due to expected lifetime pacing therapy (2). Meanwhile, conduction system pacing, including His-bundle pacing and left bundle branch area pacing (LBBAP), has emerged as a physiological alternative that directly engages the conduction system, aiming to preserve and restore ventricular synchrony (3, 4). LBBAP has demonstrated high success rates in adult populations, yielding high implantation rates and durable pacing parameters within a wide anatomical target area (3, 5).

Despite growing evidence supporting the safety and efficacy of LBBAP in adults, literature regarding its application in paediatric patients remains largely limited to case reports and small, single-centre retrospective cohorts (6–14). Furthermore, the paediatric population presents unique anatomical challenges, such as thin interventricular septa, altered cardiac orientations, and the presence of surgical patches and baffles, which may affect procedural feasibility and outcomes. The diagnostic criteria for confirming left bundle branch (LBB) capture may not be entirely applicable to children as well (15).

This systematic review and meta-analysis aimed to pool the available global evidence to evaluate the feasibility, safety, and clinical outcomes of LBBAP in paediatric patients. Additionally, we sought to identify specific patient and anatomical characteristics that predict procedural failure to better guide patient selection for this novel pacing modality.

## Methods

### Protocol design and registration

The research protocol was registered in the International Prospective Register of Systematic Reviews [PROSPERO; Registration Number: (CRD420261287485)]. Compliance with the Preferred Reporting Items for Systematic Reviews and Meta-Analyses (PRISMA) and the Cochrane guidelines was rigorously maintained. Because this study is a systematic review and meta-analysis based on previously published data and de-identified patient-level data, institutional review board approval was not required.

### Eligibility criteria

Studies were eligible if they included paediatric patients (age ≤ 18 years) who underwent attempted LBBAP, encompassing both strict left bundle branch pacing (LBBP) and left ventricular septal pacing (LVSP), and reported at least 1 of the primary or secondary outcomes of interest (procedural success rates, pacing parameters, QRS duration, left ventricular activation time [LVAT], left ventricular ejection fraction [LVEF], and/or left ventricular end-diastolic diameter [LVEDD]). Exclusion criteria were studies involving adult-only cohorts (> 18 years old), studies exclusively evaluating His-bundle pacing without LBBAP attempts, non-English publications, and case series comprising fewer than 5 patients.

### Information sources and search strategies

The search strategy included the medical subject heading terms “left bundle branch area pacing,” “LBBAP,” “conduction system pacing,” “paediatric,” “children,” “congenital heart disease,” and similar key words of interest. Searches were performed in PubMed/MEDLINE, Embase, SCOPUS and the Cochrane Library databases from database inception to 5th February 2026, restricted to English-language publications (Supplementary Table S1). To ensure a comprehensive systematic search and capture relevant grey literature, electronic database queries were supplemented by manual hand-searching of recent conference abstracts from major cardiovascular and electrophysiology societies, alongside forward and backward citation tracking of included studies. this grey literature pool explicitly incorporated our own institutional dataset (10), which had been finalised for presentation prior to the search cut-off date and subsequently formally published in June 2026.

### Study selection and data extraction

After conducting the database search and removing duplicates, 2 authors (HTL and SYK) independently screened the titles and abstracts based on predefined criteria. Selected articles were compared, with disagreements resolved by a third author (ST). Eligible studies then underwent full-text review to determine their eligibility. Included papers underwent data extraction to summarise baseline characteristics (age, weight), congenital heart disease (CHD) complexity (categorised using Bethesda Task Force criteria) (16), procedural details (fluoroscopy parameters, capture criteria), and outcomes of interest. For cohorts with multiple follow up assessments, data from the most recent echocardiogram and device interrogation were extracted for the final analysis. Where published data were aggregated or incomplete for the paediatric subgroup, corresponding authors were contacted directly to obtain raw datasets and to clarify methodological definitions.

### Procedural success and complications

Procedural success was defined across the included cohorts as the successful deployment of a pacing lead within the left bundle branch area, universally confirmed by achieving a paced right bundle branch block morphology in conjunction with study-specific electrophysiological capture criteria. Adverse events and procedural complications were evaluated strictly on a per-patient basis to determine the proportion of individuals experiencing at least one complication. Complications were categorised according to their aetiology into procedure-related events (e.g., pneumothorax, acute septal perforation) and device-related events (e.g., lead micro-dislodgement, pocket erosion). Furthermore, these were classified temporally as either acute (occurring intra-procedurally or prior to initial hospital discharge) or chronic (occurring during the outpatient follow-up period).

### Risk of bias assessment and certainty of evidence appraisal

Methodological quality and risk of bias were independently evaluated using the Joanna Briggs Institute (JBI) Critical Appraisal Checklist for Case Series. An overall risk of bias was determined for each study by applying a 75% cut-off for “Yes” responses and also the fulfilment of pre-defined critical domains. Studies were classified as “Low Risk” if they scored ≥ 75% (achieving at least 8 out of 10 “Yes” responses) and successfully met the criteria for consecutive (Domain 4 in Figure 2) and complete (Domain 5 in Figure 2) inclusion. Studies that met the 75% threshold but were rated as “Unclear” in one or more critical domains were classified as having “Some concerns”. Studies were assigned a “High Risk” rating if they failed to meet the 75% threshold or exhibited significant gaps across multiple critical or clinical reporting domains, such as the omission of procedural failures or essential demographic data. This strict threshold was applied to ensure the evidence synthesis could prioritise studies demonstrating adequate reporting transparency and selection rigour. Grading of Recommendations Assessment, Development, and Evaluation (GRADE) framework to all primary and secondary endpoints. Following established methodology, observational data received an initial certainty score of low. Final evidence grades were then determined by evaluating five domains including risk of bias, statistical inconsistency, indirectness, imprecision, and publication asymmetry.

### Meta-analyses and meta-regression

Statistical analyses were performed using JASP software (Version 0.96, University of Amsterdam, The Netherlands). Random-effects meta-analyses using the restricted maximum likelihood (REML) method were conducted to pool both continuous and dichotomous outcomes. For dichotomous procedural outcomes including overall success and complication rates, pooled event proportions and 95% confidence intervals (CIs) were calculated utilising a logit transformation. A sensitivity analysis was conducted to evaluate the primary procedural success endpoint by excluding studies classified as having a high risk of bias. For continuous variables (follow-up duration, pacing parameters, and changes in QRS duration, LVEF, and LVEDD Z-scores), pooled estimates and mean differences (MD) with 95% CIs were generated. For studies reporting continuous data as medians with interquartile ranges or minimum-maximum ranges, sample means and standard deviations were algebraically estimated utilising the validated methods described by Luo et al. (17) and Shi et al. (18, 19) For paired parameters lacking published within-patient correlation coefficients, the standard deviation of the mean difference was conservatively imputed from the baseline and post-implant standard deviations assuming a pre-post correlation coefficient of r = 0.5, in accordance with the *Cochrane Handbook for Systematic Reviews of Interventions* (20).

Inter-study heterogeneity was assessed using the Cochran's *Q*-test and the I^2^ statistic, with an I^2^ > 50% indicating substantial heterogeneity. To evaluate potential predictors of procedural outcomes, a continuous random-effects meta-regression was conducted to assess the moderating effect of body weight on procedural success. Additionally, a categorical subgroup meta-analysis was performed to evaluate the impact of congenital heart disease (CHD) anatomical complexity (simple vs. moderate/complex) on procedural feasibility. A two-tailed *p*-value of < 0.05 is considered statistically significant for all analyses. Potential publication bias and small-study effects were evaluated visually using funnel plots and quantitatively using Egger's regression test, provided a sufficient number of studies were available.

## Results

### Study selection and baseline characteristics

The systematic literature search strategy yielded a total of 1,393 records across CENTRAL, Embase, Scopus, and PubMed, with one additional record identified through grey literature sources. Following the removal of 390 duplicate citations, 1,004 unique records underwent title and abstract screening. Of these, 909 were excluded, leaving 95 articles for full-text eligibility assessment. A total of 87 studies were excluded for predefined reasons, most notably the exclusive inclusion of adult populations (*n* = 47) and incomplete paediatric data (*n* = 27). Ultimately, 8 observational cohorts meeting all eligibility criteria were included for quantitative synthesis (Figure 1) (6, 7, 12, 13).

**Figure 1:**
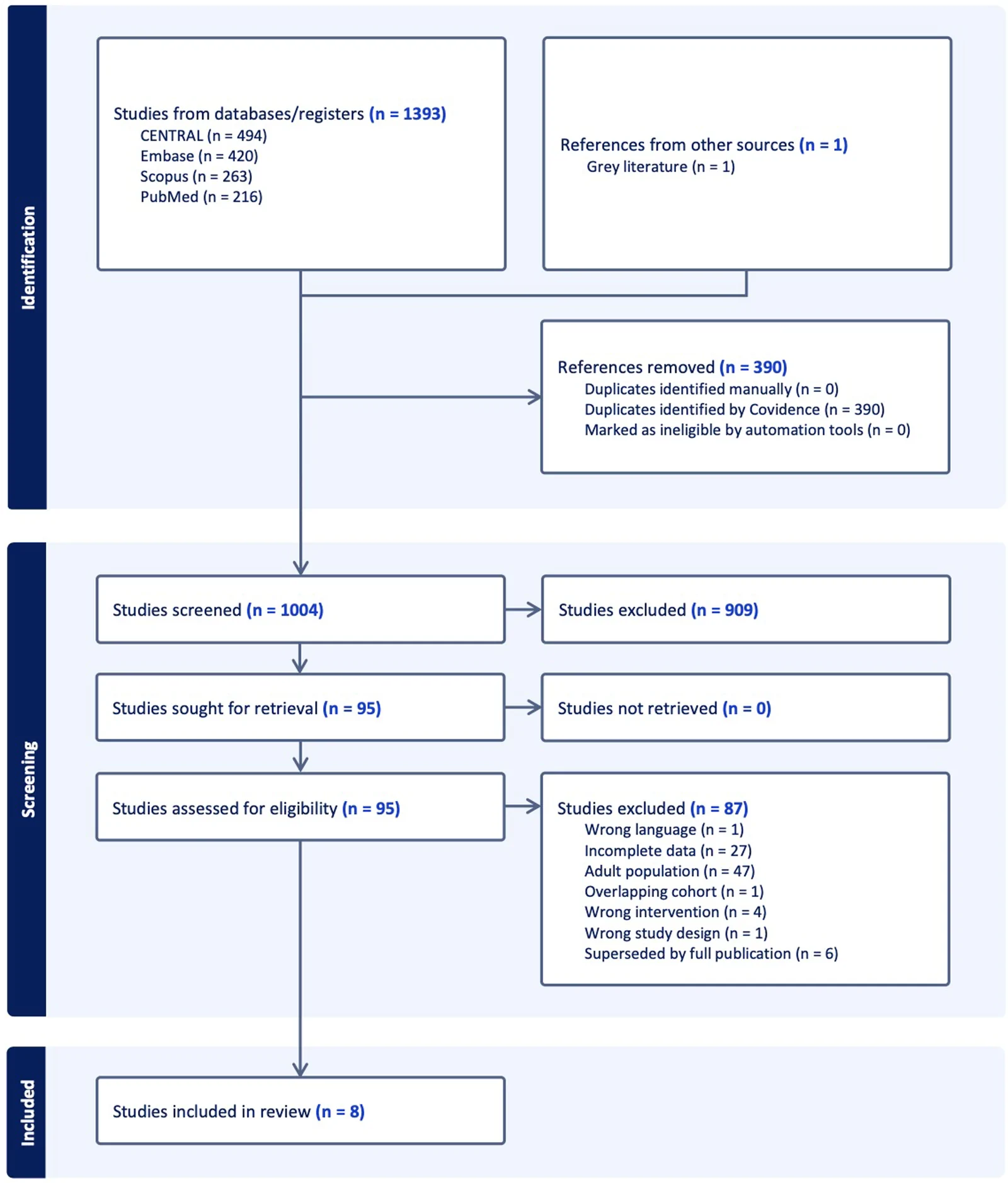
PRISMA flow diagram detailing the study selection process. Flowchart illustrating the systematic literature search, article screening, and sequential exclusion process yielding the final 8 observational cohorts included in the quantitative meta-analysis.

Across the 8 included studies, a total of 127 paediatric patients underwent attempted LBBAP. Baseline patient characteristics and demographics are summarised in Table 1. The pooled cohort reflected a wide paediatric age and weight distribution, with study-level mean ages ranging from 3.2 to 14.2 years and mean body weights ranging from 14.1 kg to 41.9 kg. Males comprised 48.2% (*n* = 55) of the population in cohorts reporting detailed sex distributions. Congenital heart disease was highly prevalent, identified in 55 patients (43.3% of the total pooled cohort). Among the 51 CHD cases with specific cardiac diagnoses reported, the majority involved simple defects (*n* = 27; 52.9%), while the remainder consisted of moderate (*n* = 15; 29.4%) and complex (*n* = 9; 17.6%) structural anatomies according to the Bethesda Task Force classification criteria (16). The underlying structural diagnoses for the remaining 4 CHD patients were not reported in the source data, precluding their classification by anatomical complexity.

**Table 1:** Baseline clinical and demographic characteristics of the included paediatric cohorts.

Study Author	Total Paediatric Patients (N)	Age (years), Mean	Body Weight (kg), Mean	Male Sex, *n* (%)	Total CHD, *n* (%)	CHD Complexity (Simple/Moderate/Complex)
Ballı et al. (6)	19	7.9	36.9	10 (52.6%)	12 (63.2%)	6/5/1
Chuang et al. (7)	9	11.2	33	5 (55.6%)	4 (44.4%)	0/1/3
Dai et al. (8)	12	8.1	28.5	5 (41.7%)	4 (33.3%)	2/2/0
Karagöz et al. (9)	18	12.3	41.5	9 (50.0%)	8 (44.4%)	6/2/0
Leung et al. (10)	16	12.7	41.9	8 (50.0%)	3 (18.8%)	0/0/3
Li et al. (11)	31	3.2	14.1	17 (54.8%)	11 (35.5%)	9/2/0
Moore et al. (12)	9	14.2	NR	1 (11.1%)	9 (100%)	4/3/2
Weinreb et al. (13)	13	NR	NR	NR	4 (30.8%)	NR/NR/NR
Total/Pooled Cohort	127	3.2–14.2^a^	14.1–41.9^a^	55 (48.2%)^b^	55 (43.3%)	27/15/9^c^

CHD, congenital heart disease; NR, Not reported.

a Represents the range of study-level reported means.

b Pooled percentage excludes the Weinreb et al. cohort, as sex distribution was not reported exclusively for the paediatric subgroup.

c Complexity classification excludes the 4 CHD patients from the Weinreb et al. cohort due to a lack of specific anatomical diagnoses.

### Methodological quality assessment

The methodological quality and risk of bias of the included studies were evaluated using the JBI Critical Appraisal Checklist for Case Series, with results summarised in Figure 2. Half of the evaluated cohorts (*n* = 4) demonstrated a low risk of bias. Two studies presented some methodological concerns due to unclear reporting on the consecutive inclusion of participants. The remaining two studies were classified as having a high risk of bias because they failed to report continuous demographic parameters, such as age and body weight, for the paediatric subgroup, which appropriately limited their inclusion in the continuous meta-regression models.

**Figure 2:**
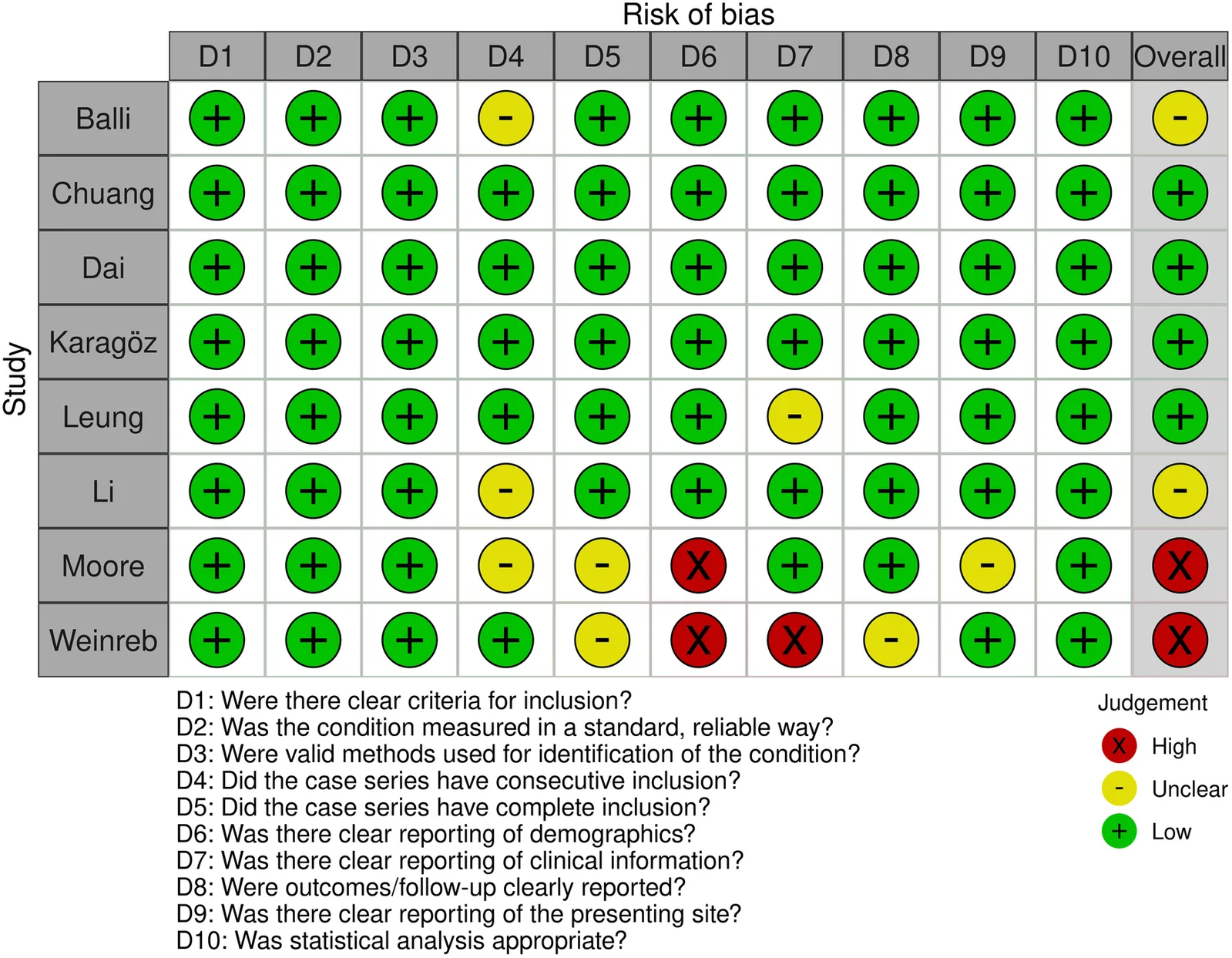
Methodological quality and risk of bias assessment. Traffic light plot summarizing the methodological quality of the included cohorts evaluated utilising the JBI Critical Appraisal Checklist for Case Series. Green circles indicate a low risk of bias, yellow indicates some concerns, and red indicates a high risk of bias in specific reporting domains (primarily driven by incomplete subgroup demographic reporting).

### Procedural success, complications, and follow-Up parameters

The pooled LBBAP procedural success rate was 80.0% (95% CI: 66.1% to 89.1%) with moderate inter-study heterogeneity (I^2^ = 52.4%, Q = 13.92, *p* = 0.053; Figure 3). This primary estimate demonstrated robustness, remaining consistent at 80.0% (95% CI: 62.3% to 90.6%; I^2^ = 61.8%, *p* = 0.031) after excluding studies with a high risk of bias in the predefined sensitivity analysis. LBBAP demonstrated an acceptable safety profile, with an aggregate complication rate of 9.7% (95% CI: 5.0% to 17.7%) with zero statistical heterogeneity (I^2^ = 0.0%, Q = 6.25, *p* = 0.511; Supplementry Figure S1). Complications were evaluated on a per-patient basis, and no individual patient in the pooled cohort experienced multiple complications. Complications specific to LBBAP were infrequent, limited to a single acute procedure-related septal perforation and one device-related micro-dislodgement that necessitated conversion to right ventricular septal pacing. The remaining adverse events reflected routine transvenous pacemaker procedural risk, consisting of one acute procedural-related pneumothorax (*n* = 1) and three chronic device -related complications including an atrial lead migration, an atrial lead diaphragmatic capture, and an erosion of the generator pocket. No late lead extraction or growth-related lead dislodgements occurred during the follow-up period.

**Figure 3:**
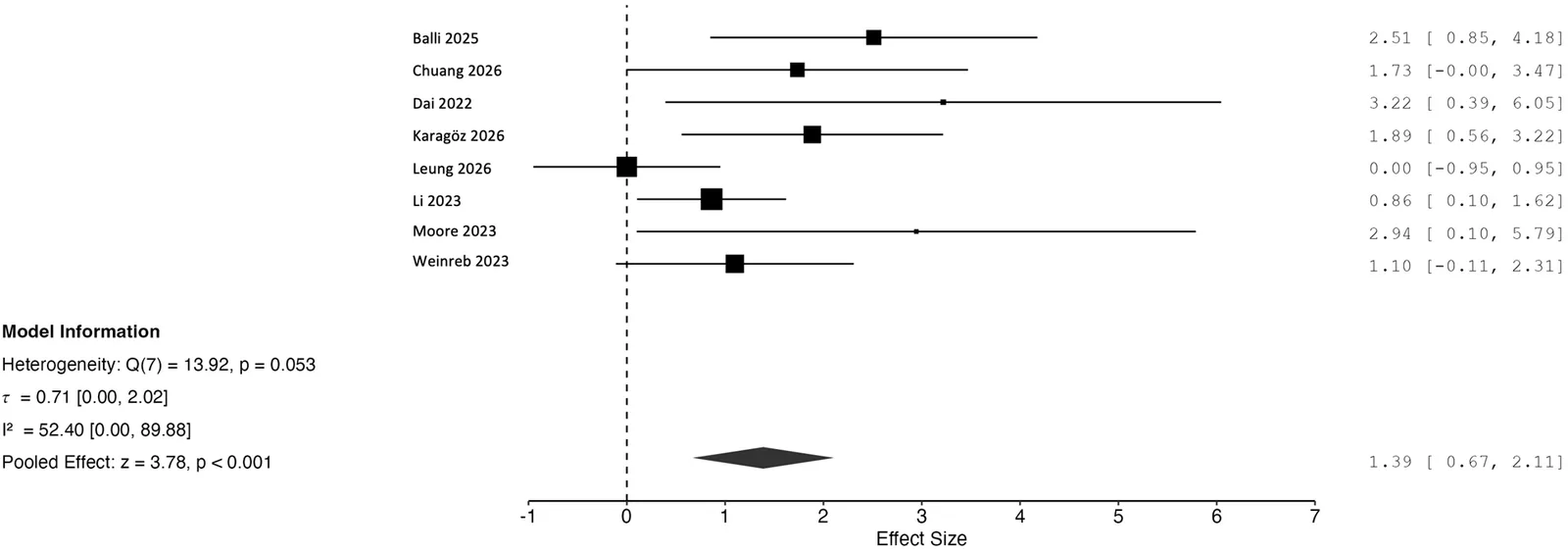
Forest plot of overall procedural success for pediatric LBBAP. Proportion meta-analysis demonstrating a pooled overall procedural success rate of 80.0% (95% CI: 66.1% to 89.1%). The size of each square is proportional to the study's statistical weight in the random-effects model utilising a logit transformation. Moderate inter-study heterogeneity was observed (I^2^= 52.4%, *p* = 0.053). *CI, Confidence Interval; LBBAP, Left Bundle Branch Area Pacing.*

The absolute values of electrical pacing parameters were evaluated at the final reported visit for each respective cohort. Over a pooled mean follow-up of 13.9 months (95% CI: 11.5–16.2 months; I^2^ = 45.5%, Q = 11.51, *p* = 0.074), electrical performance was reassuring. At these most recent device interrogations, the mean pacing capture threshold was 0.76 V (95% CI: 0.60–0.92 V), intrinsic R-wave sensing amplitude was 14.85 mV (95% CI: 13.58–16.11 mV), and pacing lead impedance was 629.7 ohms (95% CI: 560.2–699.1 ohms). All parameters exhibited significant inter-study heterogeneity (*p* < 0.05).

### Procedural techniques and delivery systems

Table 2 summarises the procedural techniques, delivery systems, and imaging modalities utilised in the included studies. Operators predominantly adapted standard adult-sized delivery systems to navigate the tighter intracardiac dimensions and altered septal orientations of the paediatric population. Among the eight included studies, delivery sheath details were reported in seven. The fixed-curve C315-His and deflectable C304-His were the most frequently utilised models, reported in 6 and 4 studies, respectively. In smaller paediatric patients or those with restricted anatomical spaces, specialised shorter curves were selectively employed. Specifically, the C315-S4 was used in 3 studies, with one centre reserving it exclusively for patients under 140 cm in height (8). The C315-S5 sheath, CPS Locator 3D, and coronary sinus delivery systems were each reported in one study. Standard fluoroscopy served as the primary imaging modality for procedural guidance across the included cohorts. Adjunctive imaging was sparse but included transthoracic and transesophageal echocardiography (10), alongside 3-dimensional mapping reported in a mixed-cohort study, without delineating its specific application in the paediatric subgroup (12).

**Table 2:** Summary of procedural techniques, imaging modalities, and electrophysiological criteria for LBBAP across included studies.

Study Author	Delivery Sheath(s) Utilised	Intra-procedural imaging	QRS morphology	Output threshold tests	LVAT/V6RWPT criteria	LBB potential criteria
Ballı et al. (6)	C315-His, C304-His	Fluoroscopy	Paced RBBB pattern/R-wave in V1	Stim-LVAT shortened abruptly or remained shortest and constant (<80 ms)		Monitored, but not strictly required for success
Chuang et al. (7)	NR	NR		Output-dependent QRS morphological transitions	<75 ms (narrow QRS) or <80 ms (LBBB/wide QRS)	Interval from pacing stimulus to V6RWPT must match intrinsic potential to V6RWPT ±10ms
Dai et al. (8)	C315-His (if height > 140 cm); C315-S4 (if height <140 cm)	Fluoroscopy, TTE		Stim-LVAT shortened abruptly or remained shortest and constant (no absolute cut-off)		Recorded in intrinsic rhythm, not necessary criteria to confirm LBB capture
Karagöz et al. (9)	C315-His	Fluoroscopy		Output-dependent QRS morphological transitions	<75 ms (narrow QRS) or <80 ms (LBBB/wide QRS)	Monitored, but not strictly required for success
Leung et al. (10)	C315-His, C304-His, C315-S4, C315-S5, CPS Locator 3D	Fluoroscopy, TEE		Output-dependent QRS morphological transitions	Absolute cutoffs intentionally excluded	Interval from pacing stimulus to V6RWPT must match intrinsic potential to V6RWPT ±10ms
Li et al. (11)	C315-S4	Fluoroscopy		Stim-LVAT shortened abruptly or remained shortest and constant (no absolute cut-off)		Monitored, but not strictly required for success
Moore et al. (12)^a^	C315-His, C304-His, CS delivery systems (Reported for mixed cohort; unspecified for paediatric subset)	Fluoroscopy, Echocardiography, RV Angiography, 3D Mapping (Reported for mixed cohort; unspecified for paediatric subset)		Abrupt Stim-LVAT change ≥10 ms or transition to selective LBBP	<75 ms (narrow QRS) or <80 ms (LBBB/wide QRS)	Recorded (20–30 ms potential-to-ventricular interval) as a marker of proximity, rather than direct proof of capture
Weinreb et al. (13)	C315-His, C304-His	Fluoroscopy		Output-dependent QRS morphological transitions	<80ms	NR

CS, coronary sinus; LBB, left bundle branch; LBBAP, left bundle branch area pacing; LBBB, left bundle branch block; LBBP, left bundle branch pacing; LVAT, left ventricular activation time; NR, not reported; RBBB, right bundle branch block; RV = right ventricular; Sti-LVAT, stimulus to left ventricular activation time; TEE, transesophageal echocardiography; TTE, transthoracic echocardiography; V6RWPT = V6 R-wave peak time.

a Note on Moore et al.: This study was a retrospective analysis conducted across 7 centres. The intraprocedural criteria and assessment of conduction system pacing were adjudicated by the individual operators alone at each respective centre. To define and classify left bundle branch capture, the operators utilised standard criteria as defined by Huang et al. (3).

### Electrophysiological criteria and the LBBAP continuum

Procedural success typically required a paced right bundle branch block (RBBB) morphology (terminal R/r' in lead V1). However, criteria distinguishing LBBP from LVSP lacked standardisation. During threshold testing, four studies utilised output-dependent QRS transitions, one required an abrupt Stim-LVAT change (≥10 ms) or transition to selective LBBP, and three required Stim-LVAT shortened abruptly or remained shortest and constant. Absolute cut-off values for the left ventricular activation time and V6 R-wave peak time also varied across the cohorts. Three studies adopted the European Heart Rhythm Association consensus criteria (21), requiring an activation time of less than 75 milliseconds for a narrow QRS and less than 80 milliseconds for a wide QRS or left bundle branch block. Two studies applied a strict limit of less than 80 milliseconds. The remaining three studies relied on abrupt activation time shortening rather than absolute cut-offs. Furthermore, left bundle branch potentials were monitored in seven studies. In two cohorts true capture was confirmed only if the interval from the pacing stimulus to the V6 R wave peak matched the intrinsic potential to V6 R wave peak interval within 10 milliseconds. The other five studies considered these potentials to be markers of proximity (Table 2). Without the ability to retroactively review individual electrograms using contemporary consensus guidelines, we cannot definitively separate true LBBP from LVSP in all instances. As a result, our findings reflect the broader clinical and procedural continuum of LBBAP.

### Predictors of procedural success

To determine if patient size or underlying anatomical complexity impacted procedural feasibility, meta-regression and subgroup analyses were performed. Continuous random-effects meta-regression demonstrated that mean baseline body weight was not a statistically significant predictor of procedural success (Q_m_ = 0.005, *p* = 0.944; Supplementary Figure S3). Furthermore, a categorical subgroup analysis evaluating the impact of anatomical complexity was performed (Supplementary Table S2). Within the pooled cohort, 55 patients had congenital heart disease, while the remaining 72 patients possessed structurally normal hearts. After excluding studies that lacked patient-level outcome data linking specific anatomies to procedural success, this analysis revealed a pooled procedural success rate of 83.6% (95% CI: 63.4% to 93.7%) in patients with simple CHD (Q_e_ = 0.29, *p* = 0.96), compared to 64.0% (95% CI: 32.8% to 86.6%) in those with moderate or complex CHD (Q_e_ = 7.14, *p* = 0.21). Although this represented a notable numerical decrease in procedural feasibility for patients with more complex underlying anatomies, an omnibus test indicated this numerical variation did not reach statistical significance (Q_m_ = 1.50, *p* = 0.22).

### Electrocardiographic and echocardiographic outcomes

Due to variations in individual study protocols, the echocardiographic reassessments used to evaluate ventricular dimensions and function were performed at different timepoints, typically captured between 3 and 12 months post-implantation. Continuous meta-analyses of paired baseline and post-implant data were performed to assess the physiological and structural impact of LBBAP. No significant average change in QRS duration was observed, with the pooled mean difference from baseline to post-implant follow-up being −2.75 ms (95% CI: −17.37 to 11.87 ms; *p* = 0.71). Significant inter-study heterogeneity was observed. (I^2^= 87.4%, Q = 34.08, *p* < 0.001) (Figure 4), which is likely driven by the highly variable baseline ventricular activation profiles across the included cohorts. Specifically, the pooling of patients with narrow intrinsic conduction, wide intrinsic escape rhythms, and pre-existing obligate right ventricular pacing.

**Figure 4:**
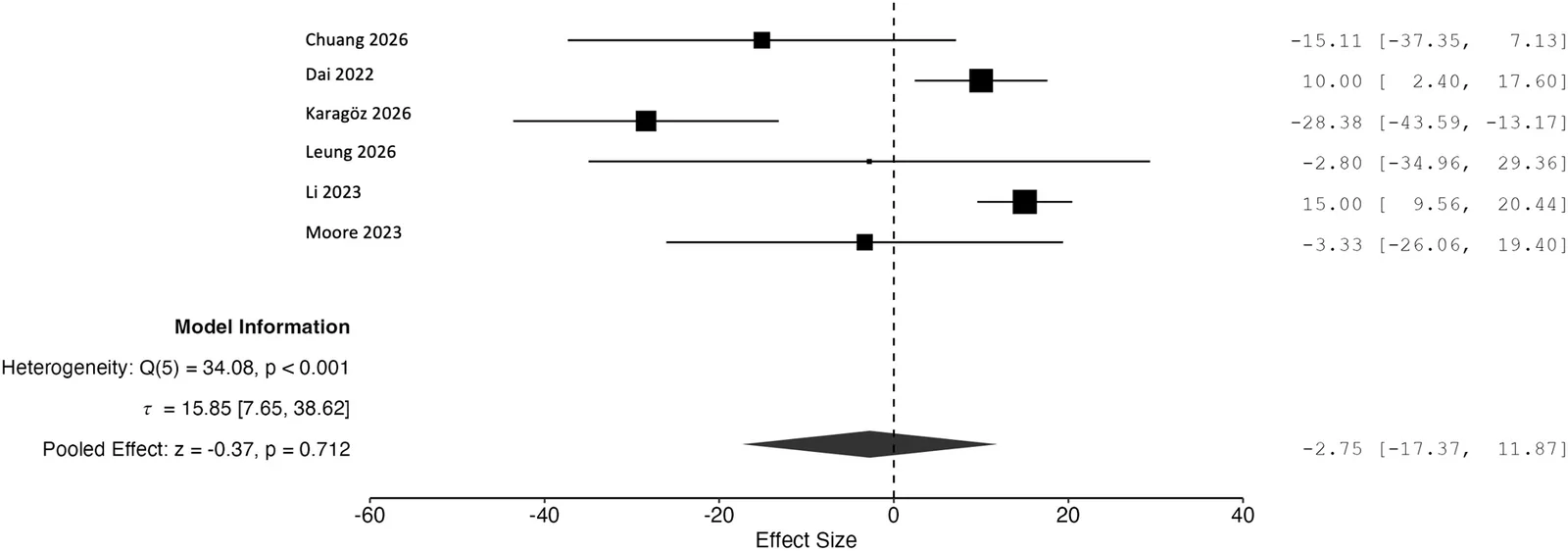
Forest plot of the meta-analysis for QRS duration. Continuous meta-analysis evaluating the overall change in QRS duration. The plot demonstrates a non-significant pooled effect for QRS duration (Mean Difference: −2.75; 95% CI: −17.37 to 11.87; *p* = 0.712). Significant inter-study heterogeneity was observed (I^2^= 87.43%, *p* < 0.001). *CI, Confidence Interval.*

Regarding the echocardiographic left ventricular functional outcomes, there was a statistically significant reduction in ventricular dimensions. The pooled mean difference in LVEDD Z-score was −1.122 (95% CI: −2.109 to −0.134; *p* = 0.026; I^2^ = 91.5%, Q = 36.74, *p* < 0.001) (Figure 5). Concurrently, global systolic function did not demonstrate statistically significant change, with a pooled mean difference in LVEF of 0.62% (95% CI: −3.25% to 4.49%; *p* = 0.754), exhibiting moderate inter-study heterogeneity (I^2^ = 55.4%, Q = 7.03, *p* = 0.071).

**Figure 5:**
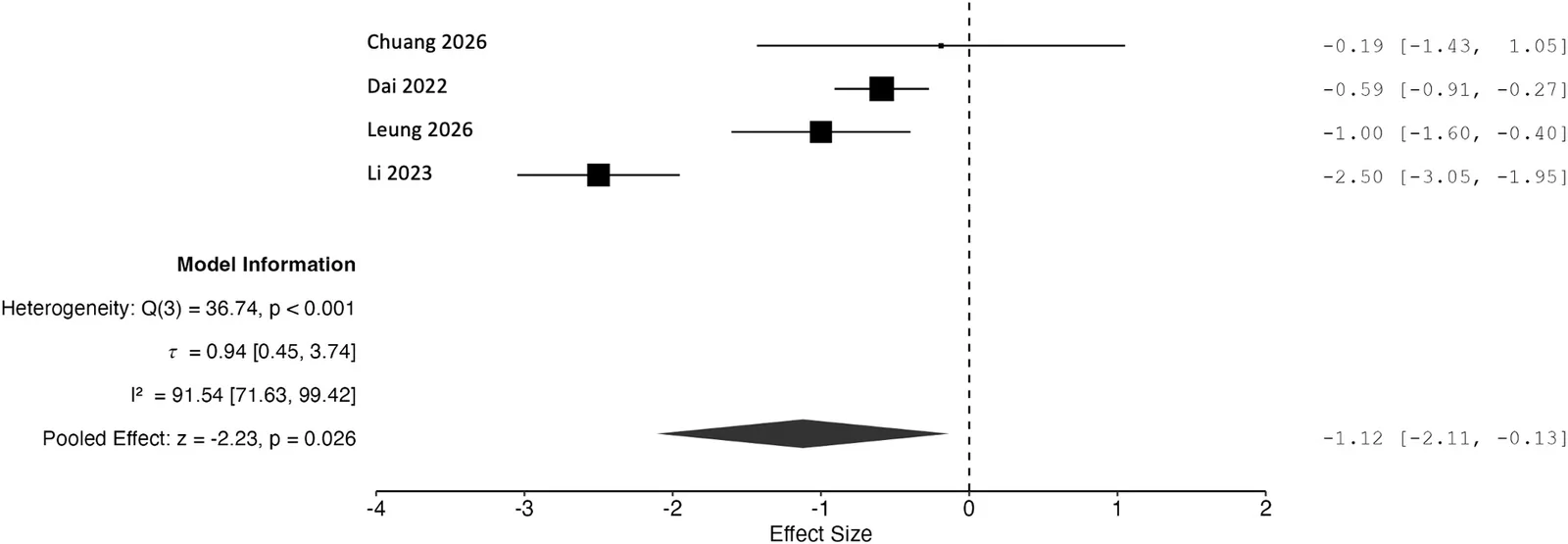
Forest plot of left ventricular end-diastolic diameter (LVEDD) Z-score change. Continuous meta-analysis comparing paired baseline and post-implant echocardiographic parameters. The plot demonstrates a statistically significant reduction in ventricular dimensions (Mean Difference: −1.122; 95% CI: −2.11 to −0.13; *p* = 0.026). *CI, Confidence Interval; LVEDD, Left Ventricular End-Diastolic Diameter; LBBAP, Left Bundle Branch Area Pacing.*

### Small-study effects, publication bias and certainty of evidence

Visual inspection of the funnel plot suggested potential asymmetry. While formal statistical testing (Weighted Regression Test t = 3.267, *p* = 0.017; Supplementary Figure S3) indicated significant asymmetry, our meta-analysis comprised fewer than 10 studies and funnel plot asymmetry tests possess low statistical power and unstable operating characteristics and therefore this assessment of publication bias is considered strictly exploratory. The suspected asymmetry observed in our analysis is typical of early literature for novel procedures, suggesting that initial institutional learning curves and technical failures might remain unpublished, whereas successful early experiences from specialised centres are preferentially reported. Application of the GRADE framework yielded a “Very Low” certainty of evidence across all evaluated procedural and electromechanical endpoints. This final appraisal reflects the inherent limitations of the foundational observational cohorts, further compounded by necessary downgrades for statistical imprecision driven by the modest pooled sample size, alongside inter-study inconsistency in continuous metrics. Due to the unreliability of the asymmetry test, suspected publication bias was not formally utilised to further downgrade the certainty of evidence.

## Discussion

To our knowledge, this study represents the first systematic review and meta-analysis evaluating the feasibility, safety, and short-to-medium-term clinical outcomes of LBBAP in the paediatric population. The principal findings suggest that LBBAP is feasible in children. Patient body weight did not significantly limit procedural success, whereas moderate-to-complex CHD was associated with numerically lower procedural success rate, though not reaching statistical significance. Moreover, LBBAP maintained baseline electrical synchrony without causing QRS prolongation usually associated with right ventricular pacing. Finally, LBBAP was associated with reduction in LVEDD Z-scores.

### Procedural feasibility and anatomical challenges

The application of LBBAP in the paediatric population remained challenging in view of smaller cardiac dimensions, thinner ventricular septa, and the anticipated mechanical strain on pacing leads due to somatic growth. Despite these factors, our pooled analysis demonstrated a satisfactory overall procedural success rate of 80.0% alongside a 9.7% aggregate complication rate. The majority of the reported adverse events were related to general access, lead, and pocket-related issues such as pneumothorax, atrial lead migration and pocket erosion (22). Such complications reflect the baseline risks of paediatric transvenous device implantation rather than hazards specific to conduction system pacing. Early-to-intermediate term follow-up data confirmed favourable electrical parameters, with low capture thresholds and satisfactory intrinsic R-wave sensing amplitudes. Preserving low capture thresholds is essential in paediatric pacing to maximise generator longevity and minimize the cumulative burden of future replacement surgeries.

An 80.0% pooled procedural success rate confirms the feasibility of paediatric LBBAP, yet this falls short of the 92.4% success documented in large adult cohorts like the MELOS registry (5). This discrepancy underscores the procedural learning curve and the anatomical constraints unique to children. Importantly, our meta-regression analysis showed that absolute body weight does not predict procedural success. However, a result lacking statistical significance in our study sample does not definitively rule out an association, and smaller patient size may still pose technical hurdles. Rather than diminishing the inherent technical difficulty of paediatric implants, this finding suggests operators are successfully adapting available equipment, such as adult-sized delivery sheaths or alternative atrial septal-oriented sheaths, to traverse restricted intracardiac dimensions (11). Conversely, our analysis observed a lower procedural success among patients with moderate-to-complex CHD when compared to simple CHD (83.6% vs. 64.0%), exposing a critical technological limitation. Although this absolute difference is highly clinically meaningful, it did not reach formal statistical significance (*p* = 0.22). This lack of statistical significance is likely driven by the limited statistical power inherent in small subgroup analyses rather than a true equivalence in procedural difficulty between these anatomical groups. Structural anomalies such as tetralogy of Fallot and atrioventricular septal defect frequently have a displaced conduction system. Implanters must manoeuvre through altered septal orientations, hypertrophied myocardium, dense fibrosis, and synthetic materials like intra-atrial baffles or ventricular patches (23). Overcoming these structural barriers may benefit from the development of highly steerable, paediatric-specific delivery sheaths. Ideally, integrating these specialised systems with routine three-dimensional electroanatomical mapping could further enhance the precision of lead deployment (24, 25).

The moderate inter-study heterogeneity observed in procedural success highlights an important clinical reality. This variance likely reflects a complex interplay of differing institutional learning curves, diverse congenital anatomies, and the challenge of adapting adult-sized delivery tools for children. Consequently, patient selection must be highly individualised, as the likelihood of a successful implant depends heavily on the patient's age, specific anatomical barriers and the centre's experience.

### Impact on ventricular function and pacing outcome

Beyond procedural success, these pooled data highlight the physiological benefits of conduction system pacing, a crucial finding for children who require lifelong pacemaker support. Chronic RV apical pacing induces asynchronous ventricular activation, triggering mechanical dyssynchrony and adverse cellular remodelling that ultimately leads to PICM (26). This risk has long been accepted as an unavoidable tradeoff for necessary pacing therapy (2).

Our findings suggest that LBBAP may mitigate this pathophysiological cascade. Baseline rhythms across the pooled cohorts were markedly heterogeneous, encompassing narrow junctional escape rhythm, wide escape rhythms, and pre-existing RV pacing. Despite this variation, the overall mean QRS duration remained statistically unchanged following implantation. Unlike the progressive electrical dyssynchrony and QRS prolongation historically associated with conventional right ventricular pacing, LBBAP did not result in significant aggregate electrical deterioration within this pooled cohort (1, 2). While varying baseline rhythm limits the utility of absolute QRS duration as a standalone marker for resynchronisation, the observed structural changes offer encouraging exploratory data. The statistically significant reduction in LVEDD Z-scores indicates that LBBAP is associated with a decrease in left ventricular dimensions during short-to-medium term follow-up. Furthermore, the lack of significant change in LVEF is not unexpected. Unlike adult cardiac resynchronisation therapy, which serves to rescue depressed systolic function, children requiring pacemakers typically have preserved baseline ventricular function (27). Taken together, these structural and functional observations suggest LBBAP may have the potential to attenuate the development of pacing-induced cardiomyopathy in children. However, further controlled longitudinal studies are required to confirm whether these findings translate into a definitive reduction in long-term heart failure risk in children.

### Electrophysiological criteria: the LBBP vs. LVSP continuum

A fundamental methodological challenge remains the precise differentiation between LBBP and LVSP. Recent adult studies indicate that LBBP confers superior electrical synchronization and clinical outcomes compared to LVSP (28). In the present systematic review, the electrophysiological definitions utilised to confirm LBB capture were markedly heterogeneous. While parameters such as output-dependent QRS transitions and shortened LVAT were commonly reported, standardised cutoffs for commonly used adult electrocardiographic criteria such as LVAT and V6-V1 interpeak intervals remain unvalidated in children. Therefore, rigid interval-based criteria are often clinically impractical. Consequently, one included study intentionally deferred absolute interval cutoffs, relying entirely on output-dependent QRS morphological transitions to verify capture (10).

Because patient-level electrograms could not be retroactively adjudicated in accordance with current international consensus guidelines (21), a definitive subgroup analysis isolating LBBP from LVSP was precluded. Consequently, the clinical outcomes reported herein reflect the broader physiological continuum of LBBAP. To accurately delineate whether the theoretically superior electrical resynchronisation of LBBP translates to long-term haemodynamic benefits over LVSP in children, future prospective paediatric registries would benefit from adhering to standardised, consensus-based capture criteria.

### Limitations

This study has several limitations. First, the observational design of the source data carries an inherent risk of selection bias and restricts the formal GRADE certainty of evidence to “Very Low.” Second, the pooled sample size of 127 patients limits the statistical power of secondary subgroup analyses. Specifically, this precluded an independent evaluation of outcomes between *de novo* implants vs. upgrades from chronic right ventricular pacing, or between patients with preserved baseline ventricular function vs. those with pacing-induced dysfunction. Third, a major limitation of the current study is the inability to perform a comparative analysis between LBBP and LVSP since diagnostic criteria remain unvalidated in children. This restricted the analysis to the broader LBBAP continuum. Consequently, it remains unknown whether the theoretically superior electrical resynchronisation of LBBP translates to better long-term clinical outcomes than LVSP in children. Fourth, the publication bias observed in the funnel plot asymmetry indicates that early institutional failures remained unpublished, implying that the 80.0% procedural success rate may slightly overestimate broad, real-world feasibility. Fifth, the short mean follow-up duration of 14 months leaves the long-term outcomes of LBBAP in growing children unaddressed, particularly the risks of growth-related lead dislodgement and future extraction complexities. Despite the inherently low certainty of available data, this study establishes the most comprehensive foundation currently available to guide paediatric LBBAP and inform the design of future prospective registries.

## Conclusion

Preliminary evidence demonstrates that left bundle branch area pacing is a feasible physiological pacing strategy in paediatric patients with an acceptable safety profile. It is associated with reduced left ventricular dimensions. Although the impacts of smaller patient size and complex congenital heart disease on procedural feasibility did not achieve statistical significance in our analysis, they likely remain important clinical considerations and technical challenges. However, reflecting the “Very Low” certainty of current observational data, these findings must be interpreted with caution. Moving forward, the adoption of standardised electrophysiological capture criteria and the establishment of prospective, longitudinal registries will be valuable to fully delineate the long-term clinical outcomes of LBBAP in the young.

## Data Availability

The original contributions presented in the study are included in the article/Supplementary Material, further inquiries can be directed to the corresponding authors.
