# Supplementary material for: Left bundle branch area pacing in children: a systematic review and meta-analysis of feasibility, safety, and outcomes
Source: Front Cardiovasc Med. 2026 Sep 16;13:1923714. doi: 10.3389/fcvm.2026.1923714 (PMC13623854; doi:10.3389/fcvm.2026.1923714)
Supplement: Supplementary file 1 [file Datasheet1.docx]

**Supplemental Appendix_Left Bundle Branch Area Pacing in Children: A Systematic Review and Meta-analysis of Feasibility, Safety, and Outcomes**

Hei-To Leung [MBBS, MRCPCH]^a^, Sabrina Tsao [MBBS, MRCPH (UK)]^a,b^, Hing-Sang Wilfred Wong [PhD]^b^, Yiu-Fai Cheung [MD]^a,b^, Sit-Yee Kwok [MBChB, MSc]^a,b^

^a^ Department of Paediatrics and Adolescent Medicine, Hong Kong Children’s Hospital, Kowloon Bay, Hong Kong. China
^b^ Department of Paediatrics and Adolescent Medicine, School of Clinical Medicine, LKS Faculty of Medicine, The University of Hong Kong, Hong Kong, China

**Correspondence:**
Dr. Sit-Yee Kwok, Cardiology Centre, Hong Kong Children’s Hospital, Department of Paediatrics & Adolescent Medicine, School of Clinical Medicine, LKS Faculty of Medicine, The University of Hong Kong.
Address for correspondence: Hong Kong Children’s Hospital, 1 Shing Cheong Rd, Kowloon Bay, Hong Kong
Email: sityeek@hku.hk
Telephone number: (+852) 35136680	Fax number: (+852) 2855-4089

Prof. Yiu-Fai Cheung, Cardiology Centre, Hong Kong Children’s Hospital, Department of Paediatrics & Adolescent Medicine, School of Clinical Medicine, LKS Faculty of Medicine, The University of Hong Kong.
Address for correspondence: Hong Kong Children’s Hospital, 1 Shing Cheong Rd, Kowloon Bay, Hong Kong
Email: xfcheung@hku.hk
Telephone number: (+852) 35136680	Fax number: (+852) 2855-4089

**Figures**

**Supplemental Figure 1. Forest Plot of Procedural Complications Associated with Paediatric LBBAP.** Proportion meta-analysis demonstrating the pooled overall procedural complication rate across the included cohorts (9.7%; 95% CI: 5.0% to 17.7%). The analysis demonstrated highly consistent safety outcomes across centres, with zero statistical heterogeneity observed (I^2^ = 0.0%, p = 0.511).

*Abbreviations: CI, Confidence Interval; LBBAP, Left Bundle Branch Area Pacing.*

**Supplemental Figure 2. Bubble Plot for Meta-Regression of Baseline Body Weight on Procedural Success.** Continuous random-effects meta-regression evaluating the moderating effect of patient size on procedural feasibility. The size of each bubble corresponds to the statistical weight of the respective study. Mean baseline body weight did not emerge as a statistically significant predictor of the logit-transformed LBBAP success rate (Q_m_= 0.005, p = 0.944).

*Abbreviations: LBBAP, Left Bundle Branch Area Pacing.*

**Supplemental Figure 3. Funnel Plot for Assessment of Publication Bias and Small-Study Effects.** Visual assessment of potential publication bias for the primary endpoint of overall procedural success. The vertical line represents the pooled effect size, while the diagonal lines define the pseudo-95% confidence limits. Although formal evaluation via a weighted regression test yielded a statistically significant result (p = 0.017), this assessment must be considered strictly exploratory. In meta-analyses comprising fewer than 10 studies, funnel plot asymmetry tests possess low statistical power and unstable operating characteristics; therefore, definitive conclusions regarding publication bias or small-study effects cannot be reliably drawn.

**Tables**

| PubMed Search Strategy | ( "Left bundle branch area pacing"[tiab] OR  "Left bundle-branch area pacing"[tiab] OR "Left bundle branch pacing"[tiab] OR  "Left bundle-branch pacing"[tiab] OR "LBBAP"[tiab] OR "LBBP"[tiab] OR "left ventricular septal pacing"[tiab] OR "LVSP"[tiab] OR "deep septal pacing"[tiab] OR "conduction system pacing"[tiab] OR "physiologic pacing"[tiab] OR "physiological pacing"[tiab]) AND ( "Pediatrics"[Mesh] OR "Child"[Mesh] OR "Adolescent"[Mesh] OR "Infant"[Mesh] OR "Infant, Newborn"[Mesh] OR "Young Adult"[Mesh] OR "Heart Defects, Congenital"[Mesh] OR "pediatric*"[tiab] OR "paediatric*"[tiab] OR "child*"[tiab] OR "adolescent*"[tiab] OR  "infant*"[tiab] OR "neonate*"[tiab] OR "newborn*"[tiab] OR "congenital heart"[tiab] OR "congenital heart disease"[tiab] OR  "CHD"[tiab] OR "congenital complete AV block"[tiab] OR "pacing-induced cardiomyopathy"[tiab] OR "PICM"[tiab])NOT ("Animals"[Mesh] NOT "Humans"[Mesh]) |
| --- | --- |
| Embase Search Strategy | (("left bundle branch area pacing" OR "left bundle-branch area pacing" OR "left bundle branch pacing" OR "left bundle-branch pacing" OR "LBBAP" OR "LBBP" OR "left ventricular septal pacing" OR "LVSP" OR "deep septal pacing" OR "conduction system pacing" OR "physiologic pacing" OR "physiological pacing").ti,ab.) AND (exp "child"/ OR exp "adolescent"/ OR exp "infant"/ OR exp "newborn"/ OR exp "congenital heart disease"/ OR ("pediatric*" OR "paediatric*" OR "child*" OR "adolescent*" OR "infant*" OR "neonate*" OR "newborn*" OR "congenital heart" OR "congenital heart disease" OR "chd" OR "congenital complete av block" OR "pacing-induced cardiomyopathy" OR "picm").ti,ab.) NOT (exp "animal"/ NOT exp "human"/) |
| Scopus strategy | TITLE-ABS-KEY ( "Left bundle branch area pacing" OR "Left bundle-branch area pacing" OR "Left bundle branch pacing" OR "Left bundle-branch pacing" OR "LBBAP" OR "LBBP" OR "left ventricular septal pacing" OR "LVSP" OR "deep septal pacing" OR "conduction system pacing" OR "physiologic pacing" OR "physiological pacing" ) AND TITLE-ABS-KEY ( pediatric* OR paediatric* OR child* OR adolescent* OR infant* OR neonate* OR newborn* OR "congenital heart" OR "congenital heart disease" OR "CHD" OR "congenital complete AV block" OR "pacing-induced cardiomyopathy" OR "PICM" ) AND NOT ( TITLE ( animal* ) OR KEY ( animal* ) ) |
| Cochrane strategy | *(left bundle branch area pacing OR left bundle branch pacing OR LBBAP OR LBBP OR left ventricular septal pacing OR LVSP OR deep septal pacing OR conduction system pacing OR physiologic pacing OR physiological pacing) AND (mh Pediatrics OR mh Child OR mh Adolescent OR mh Infant OR mh "Infant, Newborn" OR mh "Young Adult" OR mh "Heart Defects, Congenital" OR pediatric* OR paediatric* OR child* OR adolescent* OR infant* OR neonate* OR newborn* OR "congenital heart" OR "congenital heart disease" OR CHD OR "pacing-induced cardiomyopathy" OR PICM)* |

**Supplemental table 1. Full search strategy**

| **Study** | **Simple CHD Anatomy** | **Success rate** | **Moderate CHD Anatomy** | **Success rate** | **Complex CHD Anatomy** | **Success rate** |
| --- | --- | --- | --- | --- | --- | --- |
| **Balli** | VSD (n=5), ASD (n=1) | N/A | AVSD (n=4), Coarctation (n=1) | N/A | DORV (n=1) | N/A |
| **Chuang** | None | N/A | AVSD post-repair (n=1) | 4/4 (100%) | Coronary sinus type TAPVR post-repair (n=1), Criss-cross heart with VSD post-repair (n=1), d-TGA with VSD post-arterial switch (n=1) | 3/3 (100%) |
| **Dai** | VSD (n=2) | 2 / 2 (100%) | VSD + LVOT obstruction (n=1), TOF (n=1) | 2 / 2 (100%) | None | N/A |
| **Karagöz** | VSD (n=3), ASD (n=3) | 5 / 6 (83.3%) | TOF (n=1), VSD and Cor Triatriatum (n=1) | 2 / 2 (100%) | None | N/A |
| **Leung** | N/A | N/A | None | N/A | Taussig-Bing anomaly (n=1), DORV (n=1), PAVSD (n=1) | 0 / 3 (0%) |
| **Li** | VSD (n=5), ASD (n=3), PDA (n=1) | 8 / 9 (88.9%) | Complete AVSD (n=2) | 0 / 2 (0%) | None | N/A |
| **Moore** | Aortic valve disease (n=2)*, ASD/VSD (n=1), MVP (n=1) | 4 / 4 (100%) | Coarctation (n=2), AVSD (n=1) | 2 / 3 (67%) | Heterotaxy (n=1), D-TGA post-Rastelli operation (n=1) | 2/2 (100%) |
| **Weinreb** | 4 patients with CHD but details not reported | | | | | |

**Supplemental table 2 Procedural Success of LBBAP Stratified by Congenital Heart Disease Complexity** Abbreviations: ASD, Atrial Septal Defect; AVSD, Atrioventricular Septal Defect; CHD, Congenital Heart Disease; DORV, Double Outlet Right Ventricle; D-TGA, Dextro-Transposition of the Great Arteries; LBBAP, Left Bundle Branch Area Pacing; LVOT, Left Ventricular Outflow Tract; MVP, Mitral Valve Prolapse; N/A, Not Applicable; PAVSD, Pulmonary Atresia with Ventricular Septal Defect; PDA, Patent Ductus Arteriosus; TOF, Tetralogy of Fallot; VSD, Ventricular Septal Defect.

** The specific morphology of the congenital aortic valve disease was not detailed in the original source data.*
